# Deciphering the Flavor Chemistry, Processing and Quality Evaluation Methods of Milk Tea: A Comprehensive Review

**DOI:** 10.3390/foods15040681

**Published:** 2026-02-12

**Authors:** Jiayin Geng, Hongchun Cui, Yuwan Wang, Haowei Sun, Jiaqi Xu, Weiwei Wang, Feng Chen, Yun Zhao, Junfeng Yin, Jianyong Zhang

**Affiliations:** 1 State Key Laboratory of Tea Plant Germplasm Innovation and Resource Utilization, Tea Research Institute, Chinese Academy of Agricultural Science, No. 9, South Meiling Road, Xihu District, Hangzhou 310013, China; gengjiayin@tricaas.com (J.G.); wangyuwan@tricaas.com (Y.W.); sunhaowei@tricaas.com (H.S.); 947127233@stu.zafu.edu.cn (J.X.); wangwei11211@tricaas.com (W.W.); 2 Tea Research Institute, Hangzhou Academy of Agricultural Science, Hangzhou 310008, China; chc1134@126.com (H.C.); 31001@126.com (Y.Z.); 3 Urban Construction Department, Hangzhou Vocational and Technical College, Hangzhou 310018, China; teazc108@2980.com

**Keywords:** milk tea, flavor chemistry, process, quality, evaluation methods, tea, milk

## Abstract

Milk tea is a globally popular new-style tea beverage product. In recent years, the industry has achieved rapid development in terms of scale expansion and quality iteration and upgrading. The flavor quality and product stability have become the focus of attention and research hotspots in this field. The chemical foundation of milk tea flavor, processing methods, and flavor quality evaluation approaches are thoroughly elaborated. The chemical basis of tea-based, milk-based, and milk tea flavors is systematically summarized, primarily including the analysis of key flavor compounds and the interactions between tea-based and milk-based substances. Subsequently, the tea-based production methods, mixed processing techniques, and factors influencing storage and preservation of milk tea are discussed. Furthermore, evaluation methods for milk tea flavor quality, including traditional sensory evaluation and intelligent assessment techniques are systematically outlined. This review not only summarizes the recent research progress but also looks forward to the interdisciplinary work that needs to be carried out in the future. These efforts aim to provide information on the transformation from the research stage of tea milk product formulas to the development of solutions with controllable quality. Thus, they offer valuable theoretical guidance for the formation and regulation of tea milk flavor and quality as well as the development of new products. This work aims to provide theoretical insights and technical support for the translation from laboratory formulations to quality-controlled industrial solutions.

## 1. Introduction

Milk tea has gained widespread consumer popularity due to its rich taste and unique flavor profile, which is a composite beverage blending tea, milk, and sweeteners. The current market offers a diverse array of products, including traditional black tea milk foam, as well as new-style fruit tea and milk beverages. The product innovation continues to drive rapid industry expansion. According to iMedia Consulting’s “2025 China New-Style Tea Beverage Big Data Research and Consumption Behavior Survey”, China’s milk tea market reached RMB 354.72 billion in 2024 and is projected to exceed RMB 4000 billion by 2028. The number of operating milk tea stores was approximately 515,000 as of 31 August 2023, representing a growth of over 36% since the end of 2020. Despite this growth, the proportion of milk tea stores among total beverage outlets on major platforms slightly decreased to 57.7% in June 2023. To maintain competitiveness, brands prioritize flavor innovation. Surveys indicate that “new ingredient combinations” (52.44%) is the top factor influencing purchase decisions among university students, followed by “new mouthfeel” (20.73%) and “new flavor” (18.29%). Another study targeting young consumers found that approximately 26.02% consider “flavor and taste” a primary purchasing motive [1]. These findings consistently identify flavor as the core competitive advantage in the milk tea market. As a result, since 2018, brands have launched a series of innovative products, such as fruit tea, fermented rice milk tea, savory milk tea, and health-oriented milk tea. This has continuously expanded the flavor boundaries of the market to meet consumer demand for novel experiences. As the core indicator determining milk tea quality and consumer acceptance [2], flavor requires a chemical-level understanding for exploring compatibility in ready-to-drink milk tea. The formation of milk tea flavor involves complex multi-component interactions. The tea base contributes characteristic components such as tea polyphenols, catechins, and amino acids, imparting foundational notes of freshness, bitterness, astringency, and floral/fruity aromas. Components in the milk base, including milk proteins, fats, and lactose, interact with tea constituents via hydrophobic interactions and hydrogen bonding, affecting the release, perception thresholds, and stability of flavor compounds.

In recent years, analytical techniques such as gas chromatography–mass spectrometry (GC–MS) and high-performance liquid chromatography (HPLC) are widely employed. These techniques are used to characterize both volatile aroma compounds (e.g., terpenes, aldehydes) and non-volatile taste substances (e.g., esterified catechins, free amino acids) in milk tea Biomimetic sensory technologies like electronic noses and electronic tongues provide new methods for objective flavor evaluation. Although some research on milk tea has been conducted, numerous issues still require in-depth investigation: (i) The mechanisms underlying tea–milk flavor interactions are not fully elucidated, with systematic explanations lacking for the relationship between key indicators (e.g., phenolic-to-amino acid ratio, glycosidic precursors) and flavor compatibility. (ii) The impact mechanisms of processing parameters (e.g., extraction temperature and time, tea-to-milk ratio) and storage conditions (e.g., temperature, duration) on flavor stability require deeper investigation. Therefore, flavor chemistry research on milk tea is conducted to reveal the formation mechanisms of key compounds and their correlation with sensory quality. Such insights are crucial for guiding its formula optimization, process improvement, and quality standardization.

## 2. Flavor Chemical Substance Basis of Milk Tea

As a blended beverage combining tea, milk, and additives like sweeteners or nuts, milk tea flavor results from the synergistic effects of flavor compounds from the tea base, milk base, and added ingredients/processes. Variations in the types and ratios of tea, milk, and other raw materials lead to diverse flavor profiles. There have been many reports on the flavor characteristics of key flavor substances in tea and milk, but no such reports have been found for the flavor characteristics of key flavor substances in milk tea (Table 1). With the expanding milk tea market, research into its flavors has intensified, ranging from common varieties like mulberry or red date milk tea to low-sugar Yunnan black tea milk tea and regionally distinctive fermented hordeum milk tea [3,4,5,6]. Functional aspects are also explored; for instance, a lipid-lowering milk tea has been developed by combining Sanhua tea with black tea [7]. In another study, potential hypolipidemic, antihypertensive, and hypoglycemic effects have been imparted to milk tea by adding soluble dietary fiber extracted from tea stems [8]. These diverse flavors and functionalities arise from the combined regulation of raw material types, proportions, and processing techniques. A thorough analysis of milk tea flavor composition is essential. It requires identifying key flavor and taste compounds in each subsystem. This is fundamental for elucidating its flavor formation mechanisms and for guiding standardized production and quality optimization.

### 2.1. Flavor Contribution of the Tea Base

The tea base is the most critical component influencing milk tea flavor. Differences in tea cultivars and processing techniques impart distinct aromas and tastes to the tea liquor, directly shaping the final milk tea flavor. Key flavor compounds in the tea base are broadly categorized into taste compounds (Table 2) and aroma compounds (Table 3). Taste-active substances primarily include tea polyphenols (catechins, theaflavins, thearubigins, etc.), theanine, caffeine, and soluble sugars. Tea polyphenols are the main contributors to bitterness and astringency, while theanine imparts umami and sweetness, with their ratio modulating the palatability of the tea base. Aroma-active substances are dominated by terpenes, aromatic compounds, aldehydes, ketones, and heterocyclic compounds. For example, green tea contains 2-phenyl-2-butenal (sweet, honey-like) and linalool (floral) [18]; black tea features 2-pentanol (alcoholic, ripe fruity) and 2-phenyl-2-butenal (sweet, honey-like) [19]. These compounds constitute the characteristic aroma profiles of different tea bases.

#### 2.1.1. Taste Compounds

Tea polyphenols, also known as tea tannins, are the general term for polyphenolic compounds in tea leaves. Tea polyphenols are the primary taste substances and the main contributors to bitterness and astringency in tea infusion. They mainly comprise catechins, flavonoids, anthocyanins, and phenolic acids. Among these, catechins account for 70–80% of the total polyphenols and possess dual taste factors of bitterness and astringency. They mainly include epigallocatechin gallate (EGCG), gallocatechin gallate (GCG), epicatechin gallate (ECG), epigallocatechin (EGC), epicatechin (EC), and catechin (C). The astringency intensity has been ranked as ECG > CG > EG > GCG > EGC > EC > C > GC; bitterness intensity as ECG > EGCG > EC > EGC [23].

Catechins are also the main source of bitterness and astringency in green tea, with EC, EGC, ECG, and EGCG being the predominant monomeric types [24]. The bitterness intensity of EGCG is 1.5–2 times that of other catechins, while ECG is characterized by strong astringency. Together, they impart a “fresh, brisk, astringent” sensation to green tea-based milk tea. Due to enzymatic oxidation and condensation during fermentation, tea polyphenols in black tea are converted to theaflavins and thearubigins, resulting in lower polyphenol content and relatively reduced bitterness/astringency [11]. Thearubigins also contribute to the mellowness and thickness of black tea liquor, enhancing its flavor. Oolong teas exhibit varying flavors depending on their degree of fermentation. Light-fermented oolong teas, such as Light-Scented Tieguanyin, retain higher levels of EGCG, resulting in a more noticeable astringency. In contrast, heavily fermented varieties like Rich-Scented Tieguanyin undergo partial catechin conversion during roasting, a process that adds a characteristic “roasty bitterness” to the infusion [25].

Caffeine is considered one of the primary bitter substances in tea, with its bitterness intensity negatively correlated with temperature and pH. Catechins are the core contributors to bitterness and astringency in green tea infusion, with EGCG and ECG contributing over 86% of the bitterness. Furthermore, EGCG and caffeine can mutually enhance bitterness [26]. Based on the analysis of 1398 tea samples, an average caffeine content of 35.5 mg/g was found across all teas, with yellow tea having the highest content (37–47 mg/g), followed by white tea, black tea, green tea, dark tea, and oolong tea [27,28,29].

Tea contains various free amino acids exhibiting different taste attributes (Table 3), making them important taste-active compounds. Reported classifications categorize amino acid tastes into umami, sweet, bitter groups [30]. Theanine constitutes the largest proportion of free amino acids (over 40%), imparting a umami, sweet, and slightly astringent taste with a caramel-like aroma, and can mitigate the bitterness and astringency of tea while enhancing sweetness [31]. Aspartic acid and glutamic acid are the main umami amino acids; bitter amino acids mainly include isoleucine, leucine, phenylalanine, tyrosine, and valine [32]. Tasteless amino acids are defined as those with no distinct taste or only mild flavor. Examples include asparagine and cystine.

**Table 3:** Taste attributes of major amino acids in tea infusion.

Component	Taste Attributes	References
Theanine	Umami, Sweet	[33,34,35]
Asparagine	Umami, Sweet, Sour	[36]
Aspartic acid	Umami, Sour	[34,36]
Glutamic acid	Umami, Sour	[33]
Proline	Sweet, Bitter	[34,35]
Threonine	Sweet, Bitter, Sour	[35]
Cysteine	Sweet, Salty	[33,34]
Serine	Sweet, Sour	[34]
Lysine	Bitter, Sweet, Umami	[34,36]
Methionine	Bitter, Sweet	[33,35]
Tryptophan	Bitter, Sweet	[36]
Phenylalanine	Bitter	[34,35]

#### 2.1.2. Aroma Compounds

Most of the aroma in milk tea originates from the tea base used. Given that milk tea is typically consumed at lower temperatures, the perceived aroma is largely retronasal, experienced during drinking, and adds diverse flavor notes. Different tea bases have distinct aroma types related to tea category and processing characteristics. Table 4 summarizes the aroma types and key volatile compounds of different tea classes. Green tea, being non-fermented and undergoing deactivation, largely preserves the original aromatic substances of fresh leaves, resulting in a predominantly fresh, brisk aroma. Additionally, pan-fired green tea develops unique bean and chestnut aromas due to pyrazine compounds generated via the Maillard reaction during thermal processing [37]. Black tea undergoes complete withering, rolling, fermentation, and drying processes, leading to deep oxidation of polyphenols like catechins and transformation of aroma precursors such as carotenoids and amino acids [38]. This forms a rich aroma characterized by sweet, fruity, and honey notes, e.g., geraniol (rosy floral) and phenylacetaldehyde (honey-like). The unique menhuang (smoothering) process of yellow tea induces mild non-enzymatic oxidation and thermal reactions under moist heat, forming its characteristic baked rice or cooked wheat aroma. The shaking and turning process of oolong tea induces glycoside hydrolysis, producing volatile compounds like nerolidol, imparting floral and fruity aromas. Subsequent roasting promotes thermal reactions, generating pyrazines that add roasted notes. Dark tea undergoes wo-dui (pile fermentation), a microorganism-driven post-fermentation process causing profound transformation of tea components, resulting in aged, mellow, and unique fungal floral aromas. White tea processing centers on natural withering accompanied by slight auto-fermentation, yielding an elegant aroma with distinctive hao (fine hair) fragrance, along with tender and fresh floral sweetness. Aging can further develop jujube and medicinal notes.

Beyond pure tea aromas, milk tea brands often create new flavors through blending, such as by infusing the tea base with osmanthus or rose. However, research on the aroma of milk tea itself is scarce, and it remains unknown whether novel aroma compounds emerge from the interaction between tea and milk. Future research could focus on the changes in characteristic aroma compounds from different tea classes within the milk tea composite system to enable more precise flavor modulation.

### 2.2. Flavor Contribution of the Milk Base

Milk products, as an indispensable part of milk tea, have shown trends toward diversification and healthification alongside rising consumer health awareness and purchasing power. Different milk sources significantly influence the mouthfeel (creaminess, smoothness), flavor (milky aroma, sweetness), and synergistic effect with tea liquor due to variations in fat, protein content, processing techniques, and flavor compounds.

#### 2.2.1. Animal-Based Milk

Between 1978 and 2011, non-dairy creamer was the predominant dairy ingredient added to milk tea. Its primary component was hydrogenated vegetable oil. This type of oil could contain up to 60% trans fatty acids. High intake of trans fats poses significant risks to cardiovascular and cerebrovascular health. The core oils in non-dairy creamers vary, including soybean, peanut, tea seed, and coconut oils, which impart a smooth, creamy mouthfeel and rich aroma [53]. However, due to its negative health impacts and the growing health consciousness, its use has gradually been phased out.

In 2012, the launch of a fresh milk-based tea product by Heytea marked a turning point. Following this, the industry began transitioning toward using fresh milk as the primary dairy ingredient in milk tea formulations. Pure milk is categorized into pasteurized milk and ultra-high temperature (UHT) treated milk based on sterilization methods. Pasteurized milk undergoes low-temperature pasteurization, preserving most natural flavor compounds (e.g., short-chain fatty acids, carbonyl compounds) and some active proteins, resulting in a lighter taste, thinner texture, and weaker milky aroma [54]. UHT milk undergoes high-temperature treatment, inducing Maillard reactions that produce slight “cooked” or “caramelized” flavor compounds, alongside higher protein denaturation, leading to a creamier mouthfeel and stronger milky aroma. In milk tea production, UHT milk is often the preferred choice for chain brands’ standardized production due to its high stability and convenient storage. Its flavor is highly adaptable, pairing well with black teas (e.g., Assam, Ceylon) to form classic milk tea bases, and also complementing lightly fermented teas like green tea, where milk fat can coat and reduce tea astringency [55,56].

Reconstituted or modified milk differs from pasteurized and UHT milk. It involves processing adjustments or added ingredients, altering the milk’s flavor, texture, and mouthfeel to some extent. Flavored-modified milks (e.g., fruit-flavored, candy-flavored) can improve the overall flavor when blended with tea. Furthermore, to maintain milky aroma and creamy texture, concentrated milk, especially novel purified milk like “Ultra-concentrated Milk” (a freeze-concentrated milk), has gained popularity in new tea beverages [57]. Compared to fresh milk, Ultra-concentrated Milk offers a stronger milky aroma and creamier sensation. As it avoids high-temperature boiling, it maintains the fresh quality of milk, and the natural cheesy flavor produced after concentration enhances the distinctiveness of the final beverage.

#### 2.2.2. Plant-Based Milk

Since the coconut latte became extremely popular in 2021, Plant-based milk has rapidly emerged as a popular dairy alternative in recent years. It is typically produced by soaking, grinding, and homogenizing plant materials such as soybeans, almonds, oats, coconuts, or cashews. Common market varieties include soy milk, almond milk, oat milk, coconut milk, and rice milk. As no animal-derived ingredients are used, these products are naturally free of lactose and animal proteins. This makes them particularly appealing to consumers who are lactose intolerant, allergic to milk protein, or who follow vegan, environmentally conscious, or specific health-oriented lifestyles. Following the success of Luckin Coffee’s Coconut Latte in 2021, plant-based milk has become a key ingredient in milk tea innovation, introducing novel flavor combinations to the industry.

The flavor profile of plant-based milk varies significantly with its raw material and processing method, contributing distinct sensory attributes to milk tea. For example, coconut milk is rich in medium-chain fatty acids, imparting a strong natural coconut aroma. During processing, it can develop compounds such as furanones, which add a distinctive caramel-like sweetness that blends well with the robust or floral notes of tea [58,59]. Oat milk, often treated with enzymes or roasting, contains maltose from starch hydrolysis and roasting-derived aromas (e.g., alkylpyrazines). This gives it a pronounced cereal-like freshness and a mild roasted sweetness, making it especially compatible with black tea or roasted oolong tea to create a warm, full-bodied drink. Additionally, the nutty aroma of almond milk and the light taste of soy milk offer further layers of flavor diversity, expanding the creative possibilities for milk tea.

Nutritionally, plant-based milks are often rich in plant-derived components. Soy milk, for instance, contains soy protein, linoleic acid, α-linolenic acid (essential fatty acids), phospholipids, and isoflavones. Studies suggest that regular consumption may help regulate blood lipids and reduce cardiovascular risk, with soy peptides shown to have cholesterol-lowering, antioxidant, and anti-inflammatory activities [60]. However, as dairy alternatives, their nutritional profile differs fundamentally from animal milk. Most plant-based milks have a total energy content similar to low-fat or skim milk and generally contain less carbohydrate (especially in unsweetened versions). Regarding protein, soy milk is comparable to dairy milk in both quantity and quality (containing all essential amino acids). In contrast, almond, oat, and rice milks are typically lower in protein and lack a complete amino acid profile, making them insufficient as a primary protein source if consumed alone [61,62].

#### 2.2.3. Influence of Milk Protein on Milk Tea Flavor

**Figure 1:**
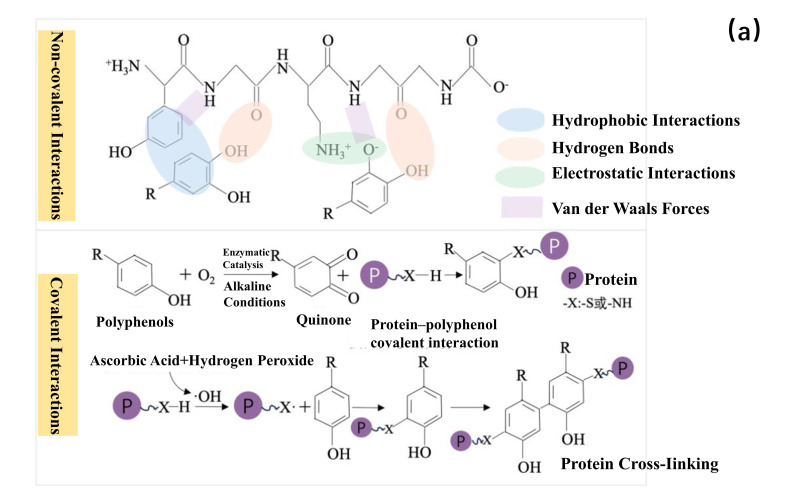

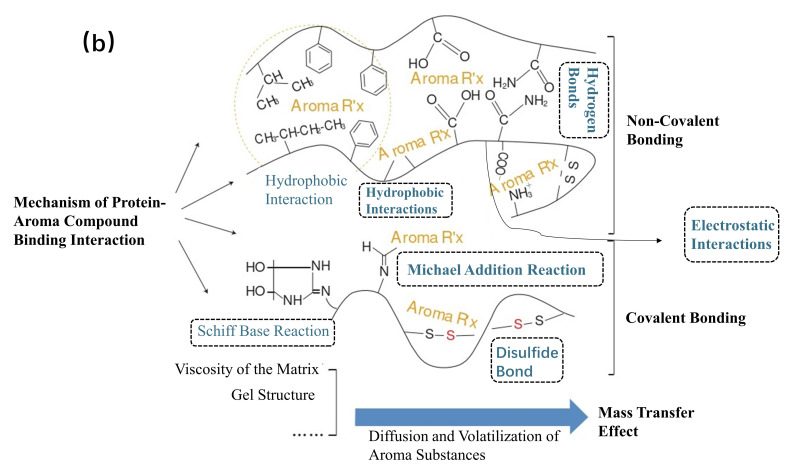
Binding Mechanism Between Proteins and Flavor Compounds, Panel (**a**) illustrates the binding mechanism between proteins and polyphenols, while Panel (**b**) depicts the binding mechanism between proteins and aroma compounds [65,66].

Milk proteins mainly comprise casein (≈80%) and whey proteins, with whey proteins further divided into α-lactalbumin (α-LA) and β-lactoglobulin (β-LG). These proteins can selectively bind with phenolic compounds (e.g., catechins) in tea infusion through non-covalent interactions such as hydrophobic interactions, hydrogen bonding, ionic bonds, and van der Waals forces (Figure 1a). Research indicates that α-lactalbumin binds with tea polyphenols via hydrogen bonds and van der Waals forces; EGCG binds with α-LA residues via hydrogen bonds and van der Waals forces; while the binding between tea polyphenols and β-LG involves hydrogen bonds and van der Waals forces [63]. The complexes formed between milk proteins and polyphenols can effectively reduce the direct contact of bitter/astringent substances like EGCG and caffeine with oral mucosa and taste receptors, thereby mitigating the bitterness and astringency of the tea infusion. Additionally, during the thermal processing of milk tea, milk proteins undergo Maillard reactions with carbohydrates such as lactose. These reactions produce volatile compounds including benzaldehyde and 2-furanmethanol, which contribute nutty, almond, and caramelized notes to the overall flavor [64]. However, excessive heating may also lead to protein denaturation, producing off-flavors (e.g., cooked flavor). Furthermore, proteins can bind with aroma compounds via non-covalent bonds like hydrophobic interactions, van der Waals forces, hydrogen bonds, and electrostatic interactions (Figure 1b), serving to retain and modulate flavor. This is because thiol groups in sulfur-containing amino acids within protein structures participate in forming disulfide bonds that stabilize protein structure, while also covalently binding with aroma compounds. The ε-amino group on lysine side chains can also bind with aldehydes via Schiff base formation [65]. Similarly, it was concluded by Anantharamkrishnan et al. that covalent bonding between milk proteins and aldehydes, thiols, and furans can occur via Schiff base formation, disulfide bond generation, and Michael addition reactions [57].

#### 2.2.4. Influence of Milk Fat and the Milk Fat-Protein Composite System on Milk Tea Flavor

Milk fat exists in the form of milk fat globules (MFGs), surrounded by a milk fat globule membrane (MFGM) composed of proteins and phospholipids. Proteins on this membrane provide abundant binding sites for polyphenols. Studies show that higher MFGM protein content correlates with stronger binding capacity to tea polyphenols and more significant astringency reduction. Moreover, homogenization to reduce fat globule size not only greatly enhances the smoothness and lubricity of mouthfeel but also increases the surface area, exposing more membrane proteins and further strengthening interactions with compounds like EGCG, thereby improving mouthfeel while enhancing astringency reduction [67]. Mo Lan’s research examined the impact of milk fat content on milk tea flavor. It showed that higher-fat milk sources produce a much stronger milky aroma, causing the milk flavor to dominate, whereas lower-fat sources allow more tea character to come through [59]. This is because higher fat content provides a richer lipophilic environment that can carry and amplify the flavor compounds from the milk source itself [68].

Additionally, milk fat plays a positive role in the colloidal stability and antioxidant activity of milk tea. The fat phase can form a hydrophobic microenvironment, protecting polyphenols from exposure to oxygen and thermal degradation. Simultaneously, polyphenols binding to lipids via hydrophobic interactions enhance their retention and stability. The protective effect of milk fat on polyphenols delays polyphenol oxidation and Maillard reactions, reduces browning index, and improves the antioxidant capacity of milk tea [64,69,70].

### 2.3. Sweeteners

Sweeteners are indispensable chemical components in the milk tea flavor system, critically influencing the overall sensory quality of the beverage primarily by providing sweetness, regulating osmotic pressure, and interacting with other flavor compounds. Commonly used sweeteners in milk tea include sucrose, high-fructose corn syrup (HFCS), and functional sugar substitutes like erythritol and steviol glycosides. Due to differences in chemical structure, metabolic properties, and sensory thresholds, these substances have varying modulating effects on the taste of milk tea (Table 5).

The influence of sweeteners on milk tea flavor primarily involves masking undesirable off-notes and enhancing existing favorable flavors and aromas. Sucrose, for instance, exerts a protective effect through hydrogen bonding between its hydroxyl groups and tea polyphenols. This reduces phenolic oxidation and inhibits o-quinone formation, thus delaying browning and flavor loss. Beyond this chemical role, sucrose also physically stabilizes the beverage by increasing its viscosity. The elevated viscosity slows down the motion of molecules like EGCG, diminishing their isomerization and degradation to preserve stable levels of flavor precursors. Furthermore, sucrose can adjust the system pH, further inhibiting polyphenol oxidase activity and reducing the generation and accumulation of bitter/astringent substances [75].

Regarding aroma, on one hand, sugars can participate in Maillard reactions during heat treatment, interacting with milk proteins or tea amino acids to generate caramel and nutty aroma compounds, enriching flavor complexity. On the other hand, the increase in aroma intensity with higher sweetener content might be because levels exceeding a certain threshold can induce protein–protein interactions, weakening protein-flavor compound interactions and thereby increasing aroma release capacity [76].

Currently, with the growing health-conscious dietary trend, an increasing number of milk tea brands opt to use sugar substitutes instead of sucrose. However, there is a lack of literature investigating the effects of different sugar substitutes and their dosage on milk tea flavor.

## 3. Processing Method of Milk Tea

**Figure 2:**
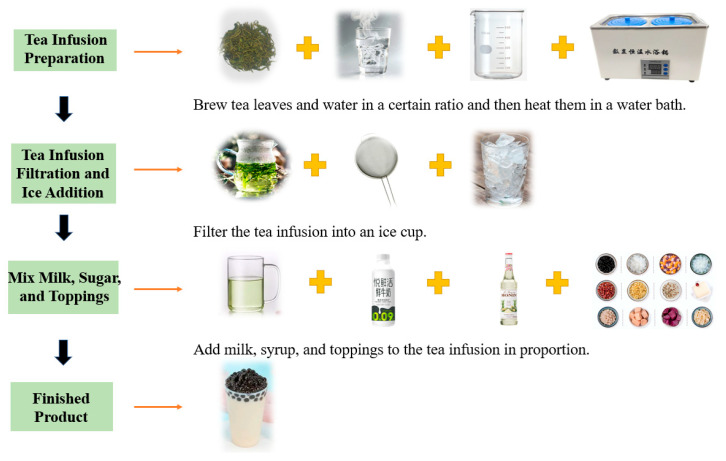
Flow Chart of milk tea Preparation.

Processing methods are the core link influencing milk tea flavor (Figure 2). Key processing parameters include extraction time, extraction temperature, tea-to-water ratio, and tea-to-milk ratio, each of which impacts the sensory flavor of milk tea.

### 3.1. Impact of Tea Soup Preparation Methods on the Flavor of Milk Tea

As the flavor foundation of milk tea, the preparation method of tea soup significantly influences its taste and aroma [77]. Tea soup is typically prepared by aqueous extraction, with extraction temperature and time being the key factors. Higher temperatures and longer durations generally intensify the bitter and astringent notes, while insufficient heat or excessive time can lead to a weak tea flavor and subdued aroma [78,79]. This can be explained from a molecular dynamics perspective: tea extraction is essentially a mass transfer process of soluble components from leaves to water. Elevated temperature accelerates molecular diffusion, promoting the dissolution of compounds such as tea polyphenols (catechins), caffeine, amino acids, and aroma precursors. Over-extraction increases the proportion of polyphenols and caffeine, resulting in a more bitter, astringent taste and a darker liquor. Excessive bitterness in the tea soup may not be adequately balanced by dairy ingredients in the final product, leading to a pronounced sweet-bitter aftertaste that compromises overall flavor. Furthermore, studies indicate that extraction temperature and time affect not only the leaching efficiency of tea solids but also the solubility of whey proteins, potentially destabilizing the milk tea system [80].

### 3.2. Tea and Milk Blending Methods

Two critical parameters govern the blending process in milk tea preparation. The first is the tea-to-water ratio. This ratio determines the strength of the tea infusion: excessive water yields a bland liquor, while insufficient water results in an overly strong, bitter, and astringent brew. Modern milk tea emphasizes the distinct taste of whole-leaf tea, necessitating an optimal ratio that highlights the tea character without excessive bitterness in the final product. Since dairy ingredients can mask much of the tea’s flavor, a relatively high proportion of tea is typically used during brewing for milk tea applications. The second key parameter is the tea-to-milk ratio. This balance directly shapes the dominant flavor profile: a high ratio allows milk to overpower the tea notes, whereas a low ratio leads to a weak and discordant taste. Therefore, blending methodology is crucial in milk tea flavor research. Traditional blending relies heavily on manual skill, where adjustments to brewing conditions and milk proportions are made based on extensive sensory experience. This often involves repeated trials to achieve the optimal taste. Table 6 summarizes the preparation methods for milk tea with different tea bases. These methods serve as a practical reference for formulation. Reflecting this need for systematic optimization, one study on Tieguanyin milk tea employed single-factor and orthogonal experiments to evaluate key variables—including extraction time, temperature, and the tea-to-milk ratio. Sensory quality evaluation was then used to determine the optimal production parameters [81].

### 3.3. Storage of Milk Tea

Milk tea, primarily prepared from milk/dairy products and tea extract/concentrate, contains the dual nutritional components of both milk and tea. The content of tea polyphenols is an important indicator for evaluating tea beverage quality [74]. After adding milk to tea, milk tea becomes a complex thermodynamically unstable system. Prolonged storage can lead to phenomena such as precipitation, flocculation, fat floating, and phase separation, affecting product appearance and taste [63,85].

Studies tracking changes over storage time have shown corresponding variations in its composition (Table 7). Research indicates that tea polyphenols are the core chemical driver of this instability. Catechins are prone to oxidation during storage. Their oxidation products not only cause browning but are also key components forming the “tea cream” precipitate [86]. Oxygen content was also indicated as a primary factor leading to tea liquor browning and polyphenol oxidation [87]. Temperature also critically affects storage stability. Higher temperatures (>25 °C) significantly accelerate the oxidative degradation of tea polyphenols, catechin isomerization, and Maillard reactions, leading to rapid decline in the content of flavor and functional components like polyphenols and amino acids, weakened antioxidant capacity, and faster precipitation formation [88,89]. Milk tea made with moderately roasted tea powder exhibited better chemical stability during storage, attributed to its lower catechin content and activity [90]. Furthermore, light exposure (especially UV) can induce and accelerate photo-oxidation reactions, directly causing tea polyphenol oxidation, vitamin degradation, and the development of unpleasant “sunlight flavor”, while also promoting liquid browning. Shu Xinyi’s research indicated that highly transparent PET bottle packaging accelerates this process, while packaging with better light-blocking properties like glass bottles or coated materials helps maintain flavor and appearance stability [91].

For fresh-made milk tea, beyond flavor, microbial growth during storage is a primary food safety concern, as these products do not undergo sterilization. A study analyzing microbial dynamics in commercially available milk tea at different temperatures found significant bacterial growth over time. For iced milk tea, the total bacterial count increased from 2.1 × 10^4^ to 2.7 × 10^5^ CFU/mL within 6 h. For hot milk tea, it increased from 7 × 10^3^ to 1.5 × 10^5^ CFU/mL over the same period [87]. Although specific standards for milk tea are lacking, the clear trend of increasing microbial counts with storage time strongly suggests that fresh-made products should be consumed promptly.

## 4. Sensory Evaluation Methods for Flavor Quality of Milk Tea

### 4.1. Sensory Evaluation

**Figure 3:**
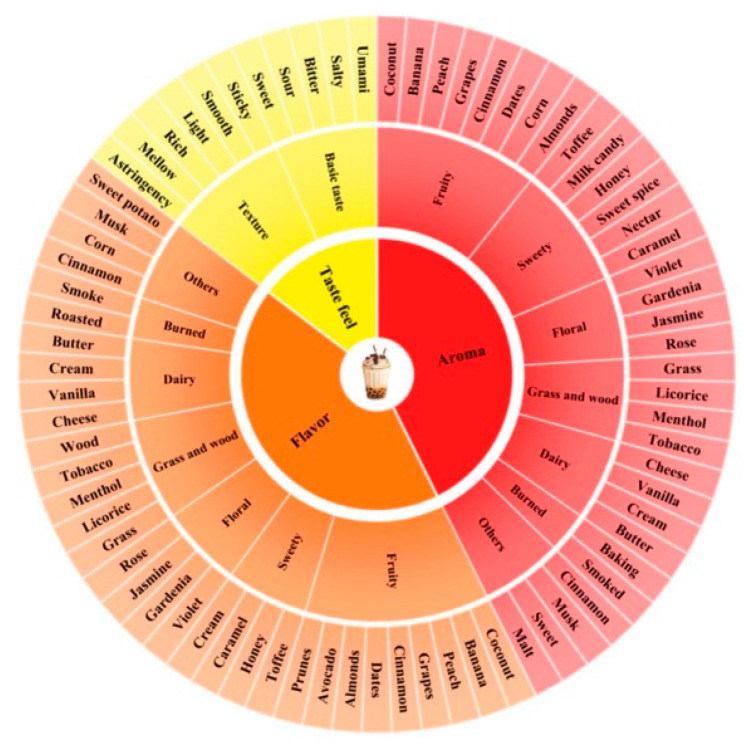
Milk tea Flavor Wheel [94].

Sensory evaluators are typically categorized into five types: expert, consumer, naive, experienced, and trained. Expert and consumer types are most widely applied in research [81,93]. Before forming a sensory panel, evaluators need to be screened and undergo systematic training. The core objective is to establish a unified cognitive standard among evaluators for the intensity of core attributes like color, aroma, and taste in milk tea, reducing individual subjective bias. During evaluation, milk tea needs to be described using specific terms. However, as there is currently no unified standard for milk tea sensory descriptors domestically or internationally, some scholars adopt tea sensory descriptors for milk tea; others opt for custom-defined descriptors [94]. The sensory descriptors, along with their corresponding aromas or tastes, are then organized into a flavor wheel for visual presentation (Figure 3).

### 4.2. Traditional Sensory Analysis Methods

#### 4.2.1. Difference Testing

Difference testing involves comparing the sensory characteristics (e.g., color, odor, taste) of two or more products to determine if a difference exists [95]. The most commonly used methods include paired comparison test, triangle test, and duo-trio test.

#### 4.2.2. Descriptive Analysis

Quantitative Descriptive Analysis (QDA) is a method for assessing the sensory attributes (e.g., appearance, odor, texture) of a product and precisely quantifying their intensity. It requires rigorous training of evaluators beforehand to minimize personal subjective perceptions and scoring errors, followed by constructing flavor profiles using Principal Component Analysis (PCA) [96]. Quantitative Descriptive Analysis (QDA) has been effectively applied to elucidate key drivers of consumer preference and to differentiate flavor profiles in tea beverages. For instance, in peach-flavored tea, QDA combined with preference mapping identified sweetness and fruity aroma as primary liking factors [97]. Furthermore, QDA has been employed to systematically analyze and document the flavor variations resulting from the use of different tea varieties as a base [93].

#### 4.2.3. Affective Testing

Affective testing measures evaluators’ emotional responses (e.g., degree of liking, preference, acceptance) towards a product. Unlike the previous methods, its focus is on which product is preferred and to what extent. Tapioca pearl size and sweetness level were found to be key factors for Asian consumers in choosing milk tea, a conclusion reached by Ardvin Kester S. Ong et al. using conjoint analysis [98].

Affective testing typically involves consumer-type subjects and generally requires a larger sample size. However, it is susceptible to interference from external factors and requires measures like blind testing to improve objectivity.

### 4.3. Digital Quality Evaluation Technologies for Milk Tea

Traditional sensory evaluation methods are susceptible to the influence and limitations of individual sensory subjectivity, such as personal preferences and mood [99]. Consequently, an increasing number of neuroscience-derived methods are being integrated into flavor analysis, providing a more comprehensive understanding.

#### 4.3.1. Electronic Tongue Technology

**Figure 4:**
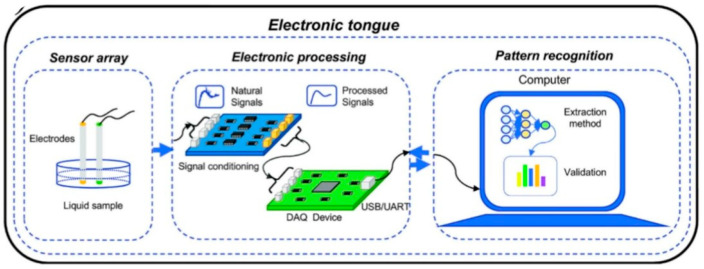
Sensing mechanisms and signal flow in electronic tongues [103].

Operating as a taste sensor, the electronic tongue (Figure 4) works by using a multi-sensor array to detect sample signals and then processing them via pattern recognition. This approach, enhanced by expert system learning, enables it to conduct qualitative or quantitative analyses, thereby simulating human taste perception [100]. The electronic tongue has been applied as an effective tool for taste analysis in tea beverage development. An objective analysis of umami substances in tea can be achieved through the simulation of human taste [101]. In another, its integration with GC–MS facilitated the optimization of a reduced-salt milk tea formula, identifying yeast extract as a key component for improving overall flavor balance [102].

#### 4.3.2. Electronic Nose Technology

**Figure 5:**
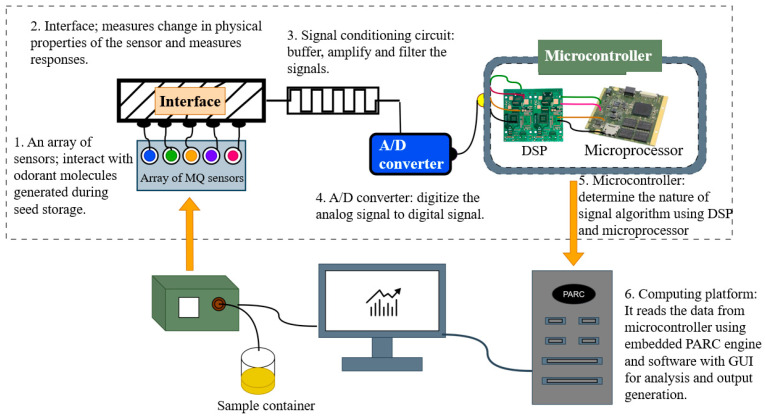
Working Principle of Electronic Nose.

The electronic nose, as an olfactory-mimicking device, also typically consists of multiple sensor arrays used to detect odors by analyzing generated signals to differentiate complex samples [104] (Figure 5). The electronic nose has demonstrated versatile applications across food quality control. In agricultural products, a customized e-nose system was developed to assess strawberry freshness, successfully discriminating between fresh and rotten samples based on their volatile profiles [105]. For tea analysis, the integration of e-nose with HS–SPME–GC–MS enabled the identification of six key aroma compounds (e.g., benzyl mercaptan) and effectively differentiated five high-aroma black tea varieties [106]. Similarly, in dairy evaluation, e-nose technology combined with sensory analysis provided a comprehensive characterization of flavor distinctions among different brands of UHT skimmed milk [107].

#### 4.3.3. Oral Tribology Evaluation Technology

Oral tribology evaluation is a technique that assesses mouthfeel sensations (e.g., smoothness, roughness, sliminess) generated by food (like jelly, cheese) or oral products (like toothpaste) in the mouth by simulating physical behaviors such as friction, adhesion, and lubrication [108]. In essence, it is used to describe and simulate the texture of food, such as the silkiness of chocolate, the crispiness of chips, the softness of bread, and the prickling sensation of carbonated drinks. The application of oral tribology has yielded actionable insights for texture science. It has been employed to establish evaluation methods and predictive models for the smoothness of complex beverages like milk tea, directly linking formula variables to sensory outcomes [109]. Furthermore, through model emulsion studies, it has identified key factors affecting oral lubrication, demonstrating that smaller oil droplets improve lubricity while saliva addition alters frictional properties [110].

## 5. Conclusions and Future Perspectives

With the continuous development of the milk tea industry and innovation in product categories, the novelty of flavor and the stability of quality are core competitive factors for attracting consumers. Although a preliminary sensory evaluation system for describing and quantifying milk tea flavor characteristics has been established, significant shortcomings remain. Specifically, evaluation terminology, standards, and procedures lack industry-wide uniformity, leading to poor comparability of results between different evaluators and affecting the accuracy and consistency of flavor descriptions. Future research should focus on establishing a standardized, systematic set of sensory evaluation descriptors and methods for milk tea. This involves creating a defined lexicon of descriptors and corresponding assessment protocols. A specialized sensory panel must be trained accordingly. The integration of modern analytical techniques, such as gas chromatography–mass spectrometry (GC–MS), will enable the qualitative and quantitative analysis of key flavor compounds. Together, these approaches will provide objective data to support and validate subjective sensory assessments. Secondly, regarding flavor compatibility, the synergistic interaction between tea base and milk base flavors and their compositional interplay is key to flavor modulation. Complex interactions occur between compounds like tea polyphenols, amino acids, and caffeine in tea and components like proteins and fats in milk. For instance, the binding of phenolic compounds with proteins can influence astringency and creaminess, while fats can adsorb and carry aroma molecules. However, there is currently a lack of systematic, in-depth exploration into the microscopic mechanisms of these component interactions, particularly how they collectively affect the release and perception of key aroma compounds. Therefore, future work should focus on investigating the molecular interaction mechanisms of key flavor compounds within the tea–milk mixed system to provide data support and scientific basis for milk tea flavor compatibility.

Furthermore, stability is the guarantee of flavor quality. During a product’s shelf life, flavor compounds can degrade or transform due to oxidation, light exposure, temperature fluctuations, and other factors, leading to flavor deterioration. Simultaneously, microbial growth not only causes undesirable flavor changes but also poses direct food safety risks. Therefore, while optimizing flavor, stability must also be regulated to ensure both flavor quality and food safety. In terms of intelligent technologies for milk tea, the integration of artificial intelligence and big data allows for the development of predictive flavor models. These models enable intelligent formula design and precise quality control, driving the industry’s digital and intelligent transformation. Future research should focus on several key areas: establishing a standardized evaluation system, elucidating the interaction mechanisms between tea and milk, innovating stability technologies, and developing healthier products. Advancements in these areas will guide the milk tea industry toward higher-quality development.

## Figures and Tables

**Table 1:** Flavor characteristics of key flavor substances in tea base, milk base and milk tea.

**Category**	**Flavor Factor**	**Key Compound**	**OAV**	**TAV Range**	**Threshold**	**Sensory Description**	**References**
Tea base	Taste	EGCG	\	0.1569–4.1883	220 μmol/L	Distinct bitterness and strong astringency	[9,10]
		EGC	\	\	1630 μmol/L	Distinct bitterness and strong astringency	[10,11]
		GCG	\	0.1927–1.3983	330 μmol/L	Distinct bitterness and strong astringency	[9,10]
		GC	\	0.0707–1.0770	1630 μmol/L	Distinct bitterness and strong astringency	[10]
		ECG	\	0.2734–4.6205	180 μmol/L	Distinct bitterness and strong astringency	[10]
		EC	\	0.0633–0.5347	860 μmol/L	Distinct bitterness and strong astringency	[10]
		CG	\	0.0633–0.5347	170 μmol/L	Distinct bitterness and strong astringency	[10]
		C	\	0.0815–0.6631	860 μmol/L	Distinct bitterness and strong astringency	[10]
		Aspartic Acid	\	1.5867–5.0336	30 mg/L	Sour	[10]
		Glutamic Acid	\	0.3249–3.2039	50 mg/L	Sour	[10]
		Serine	\	0.0094–0.1673	1500 mg/L	Sweet, Sour	[10]
		Asparagine	\	0.0189–0.1328	1000 mg/L	Sour	[10]
		Glycine	\	0.0142	1300 mg/L	Sweet	[10]
		Threonine	\	0.0017–0.0099	2600 mg/L	Sweet, Bitter, Sour	[10]
		Proline	\	0.0030–0.0221	3000 mg/L	Sweet, Bitter	[10]
		Theanine	\	0.1556–0.8456	1045.2 mg/L	Astringent, Refreshing	[10]
		Alanine	\	0.0094–0.1329	600 mg/L	Sweet	[10]
		Arginine	\	0.0010–0.3327	500 mg/L	Bitter	[10]
		Valine	\	0.0469–0.1984	400 mg/L	Sweet, Bitter	[10]
		Methionine	\	0.0000–0.0585	300 mg/L	Bitter	[10]
		Cysteine	\	0.5758–5.8492	20 mg/L	Salty	[10]
		Isoleucine	\	0.0122–0.0861	900 mg/L	Bitter	[10]
		Leucine	\	0.0376–0.1586	1000 mg/L	Bitter	[10]
		Histidine	\	0.0357–0.2397	200 mg/L	Sweet	[10]
		Phenylalanine	\	0.0119–0.0799	900 mg/L	Bitter	[10]
		Lysine	\	0.2806–0.8953	500 mg/L	Sweet, Bitter, Umami	[10]
		Tyrosine	\	0.0132–0.2313	2342.7 mg/L	Tyrosine	[10]
	Aroma	Phenylacetaldehyde	1.71	\	4 μg/L	Fresh, hyacinth note	[12,13]
		Benzyl alcohol	15.79	\	0.4 μg/L	Fatty note	[12]
		Heptanal	<1	\	10 μg/L	Grassy note	[12]
		Decanal	11.71	\	6 μg/L	Floral, fruity, sweet note	[12]
		Methyl palmitate	<1	\	1000 μg/L	Fatty, waxy note	[12]
		Nonanal	44.87	\	1 μg/L	Rose, citrus fruity note	[12]
		α-Ionone	58.24	\	0.4 μg/L	Violet, woody note	[12]
		1-Octen-3-ol (Matsutake alcohol)	15.89	\	1 μg/L	Mushroom note	[12]
		Cedrol	49.01	\	0.5 μg/L	Woody, aged note	[12]
		Methyl linoleate	<1	\	450 μg/L	Oily note	[12]
		Linalool	28.25	\	0.0015 μg/L	Floral, sweet fruity note	[12]
		Dihydroactinidiolide	<1	\	500 μg/L	Coumarin, woody, fruity note	[12]
		Methyl salicylate	-	\	40 μg/L	Wintergreen leaf note	[12]
		2-Pentylfuran	-	\	6 μg/L	Fruity, green, metallic note	[12]
		α-Terpineol	<1	\	300 μg/L	Sweet floral, fresh, woody note	[12]
		trans-Linalool oxide (furanoid)	2.79	\	6 μg/L	Aromatic pine nut note with slightly sweet citrus flavor	[12]
		Linalool oxide I (cis)	9.1	\	6 μg/L	Floral, sweet, woody note	[12]
		Geraniol	<1	\	32 μg/L	Pineapple note	[12]
		Methyl octanoate	<1	\	60 μg/L	Wine, fruity, sweet orange note	[12]
Milk base	Taste	Milk Fat	\	\	N/A	Provides richness, smoothness, and natural creamy flavor	[14]
		Lactose	\	\	N/A	Mild, subtle sweetness	[15]
	Aroma	δ-Decalactone	\	\	2.50–410.00 μg/kg	Creamy aroma	[16]
		Diacetyl	\	\	12 μg/kg	Characteristic natural butter aroma	[17]
		Methyl mercaptan	\	\	0.02–2.1 μg/L	steamed note, cabbage-like note	[17]
milk tea	Taste	\	\	\	\	\	\
	Aroma	\	\	\	\	\	\

Note: OAV (odor activity value) and threshold are two key parameters used to evaluate the intensity and importance of the taste and aroma of food. The calculation formula is the concentration of the substance divided by the threshold. The higher the value, the greater the contribution of the substance to the taste and aroma of the food. TAV (Taste Activity Value). It is a key indicator for assessing the contribution intensity of a taste substance to the overall flavor of a food. Its calculation formula is: TAV = concentration of the substance in the food/taste threshold.

**Table 2:** Main taste compounds in tea bases.

**Category**	**Key Components**	**Taste Attributes**	**References**
Tea Polyphenols	Eight catechin monomers (EGC, GC, EC, C, EGCG, ECG, GCG, CG)	Bitter, Astringent	[9,20]
	Oxidized polymer (theasinensin, theaflavins, thearubigins)	Bitter, Astringent	[21]
	Flavones, Flavonoid glycosides (quercetin, kaempferol, myricetin)	Astringent	[9]
	Phenolic acids (gallic acid, p-coumaric acid, chlorogenic acid, theogallin)	Sour, Astringent	[20,22]
Alkaloids	Theobromine, Theophylline, Caffeine	Bitter	[21]
Amino Acids	Umami amino acids (L-Th, Glu, Asp)	Umami	[9,20]
	Sweet amino acids (L-Th, Gly, Ala)	Sweet	[22]
	Bitter amino acids (Arg, Leu, Phe)	Bitter	[9,21]
Soluble Sugars	Fructose, Glucose, Maltose, Sucrose, Galactose, Xylose	Sweet	[22]

**Table 4:** Aroma types and key volatile compounds of different tea classes.

Tea Class	Aroma Type	Aroma Components	References
Green Tea	Fresh	Methyl salicylate, Nonanal, (Z)-3-Hexenal	[39,40,41,42]
	Tender	Nonanal, Hexanal, Heptanal	
	Chestnut	Linalool, Cedrol, Heptanal	
	Beany	Phenylacetaldehyde, Methyl salicylate, 2-Ethyl-3,5-dimethylpyrazine	
	Grassy	(Z)-3-Hexen-1-ol,(E)-4-Heptenal, Hexanoic acid	
	Floral	Cyclofenchene, Geranyl linalool, (E)-2-Decenal	
Black Tea	Fruity	Myrcene, Limonene, Nerol	[42,43]
	Floral	Linalool, Geraniol, cis-Jasmone	
	Honey	Phenylacetaldehyde, Phenylethyl alcohol, Octanal	
Oolong Tea	Floral	Nerolidol, Indole, Geraniol	[44,45]
	Fruity	Methyl salicylate, Benzyl alcohol, Ethyl acetate	
	Roasted	Pyrazines, Pyrroles, Furans	
Dark Tea	Aged	1,2-Dimethoxybenzene,1,2,3-Trimethoxybenzene,4-Ethyl-1,2-dimethoxybenzene	[46,47,48]
	Mellow	α-Cedrol, Guaiacol, β-Ionone	
	Woody	Eucalyptol, Longifolene, β-Cedrene	
	Fungal Floral	1-Octen-3-ol,3-Octanone, Hexyl hexanoate	
White Tea	Hao Fragrant	cis-3-Hexenol,1-Octen-3-ol, Benzyl alcohol	[49,50]
	Tender	Hexanal, (Z)-3-Hexenal, (E)-2-Hexenal	
	Floral	Linalool, Phenylethyl alcohol,α-Farnesene	
	Nutty	2-Pentylfuran, Pyrazines, Methoxybenzenes	
Yellow Tea	Nutty	2,5-Dimethylpyrazine, 2-Acetylfuran, Pyrroles	[51,52]
	Tender	Hexanal, (Z)-3-Hexenal, (Z)-3-Hexenol	
	Sweet	Linalool, Maltol, 2-Methylbutanal	

**Table 5:** Chemical and sensory properties of common sweeteners in milk tea.

Sweetener	Main Component(s)	Relative Sweetness (Sucrose = 1)	Source	Taste Profile	References
Sucrose	Disaccharide (Glucose + Fructose)	1	Natural	Balanced, mellow sweetness; mild onset, moderate aftertaste.	[71]
HFCS	Mixture of Glucose and Fructose	1.5–1.8	Natural	Direct sweetness, pronounced “cooling sweet sensation”; may impart slight sour aftertaste at high usage levels.	[71]
Erythritol	Sugar alcohol	0.6–0.8	Natural	Clean, mild sweetness with slight cooling sensation; strong bitter/astringent aftertaste and slight green tea note, differing significantly from sucrose.	[71,72]
Steviol Glycosides	Terpene glycosides	200–300	Natural	Intense, powerful sweetness (high-potency); immediate sweet onset; very strong bitter/astringent, licorice-like aftertaste and green tea note, most different from sucrose.	[71]
Xylitol	Sugar alcohol	0.8–1	Natural	Cooling sensation, full-bodied sweetness, closest flavor similarity to sucrose.	[72,73]
Aspartame	L-Aspartyl-L-phenylalanine methyl ester	180–220	Artificial	Pure sweetness, fruity note, basic sweetness similar to sucrose; heavier sweet aftertaste, overall flavor difference less than steviol glycosides.	[74]

**Table 6:** Preparation Technology of milk tea with Different Tea Bases.

Tea Base	Extraction Temperature (°C)	Extraction Time (min)	Tea-to-Water Ratio	Tea-to-Milk Ratio	References
Black Tea	85	10	1:30	10:3	[6]
Dark Tea	95	20	1:40	10:1	[82]
Oolong Tea	90	20	1:50	6:1	[81]
Yellow Tea	89.8	27.04	1:39.2	2:1	[83]
Green Tea	85–90	7	1:30–1:50	2:1	[84]

**Table 7:** Effects of Different Storage Times on milk tea.

Time	4 °C				25 °C				Light Exposure				References
	Tea Polyphenols (mg/kg)	Protein (%)	Oil Floating Rate (%)	Precipitation Rate (%)	Tea Polyphenols (mg/kg)	Protein (%)	Oil Floating Rate (%)	Precipitation Rate (%)	Tea Polyphenols (mg/kg)	Protein (%)	Oil Floating Rate (%)	Precipitation Rate (%)	
Initial	1160	1.18	0.65	0.40	1160	1.18	0.65	0.4	1160	1.18	0.65	0.40	[92]
3 Months	1156	1.17	1.05	0.43	1100	1.10	1.25	0.53	1070	1.07	1.41	0.77	
6 Months	1150	1.10	1.79	0.63	1090	1.12	2.69	0.73	1040	1.10	2.89	0.93	
9 Months	1154	1.08	2.93	0.70	1070	1.06	3.33	0.99	1030	1.08	3.83	1.32	
12 Months	1130	1.07	3.19	1.01	1020	1.00	4.29	1.37	980	0.97	4.54	1.61	

## Data Availability

No new data were created or analyzed in this study.
